# Editorial: Hypoxia and Cardiorespiratory Control

**DOI:** 10.3389/fphys.2021.820815

**Published:** 2021-12-22

**Authors:** Yasumasa Okada, Julian F. R. Paton, José López-Barneo, Richard J. A. Wilson, Nephtali Marina, Mieczyslaw Pokorski

**Affiliations:** ^1^Clinical Research Center, Murayama Medical Center, Tokyo, Japan; ^2^Manaaki Manawa—The Centre for Heart Research, Department of Physiology, Faculty of Health and Medical Sciences, University of Auckland, Auckland, New Zealand; ^3^Instituto de Biomedicina de Sevilla (IBiS), Hospital Universitario Virgen del Rocío, Consejo Superior de Investigaciones Científicas (CSIC), Universidad de Sevilla, Seville, Spain; ^4^Departamento de Fisiología Médica y Biofísica, Facultad de Medicina, Universidad de Sevilla, Seville, Spain; ^5^Centro de Investigación Biomédica en Red sobre Enfermedades Neurodegenerativas (CIBERNED), Madrid, Spain; ^6^Department of Physiology and Pharmacology, Hotchkiss Brain Institute and Alberta Children's Hospital Research Institute, University of Calgary, Calgary, AB, Canada; ^7^Division of Medicine, University College London, London, United Kingdom; ^8^Institute of Health Sciences, Opole University, Opole, Poland; ^9^Faculty of Health Sciences, The Jan Dlugosz University, Czestochowa, Poland

**Keywords:** intermittent hypoxia, sleep apnea, pulmonary hypertension, sympathetic excitation, astrocyte, TRPA1, carotid body, plasticity

## Introduction

To maintain adequate oxygen levels in the body, which is essential for a healthy life, the respiratory and cardiovascular systems play vitally important roles. When the oxygen content is insufficient, i.e., when hypoxia is loaded, respiratory and cardiovascular systems respond to restore, compensate, or adapt to hypoxia, e.g., by increasing ventilation and blood flow to maintain oxygen transport to vital organs. Traditionally, it has been thought that hypoxia is detected solely by carotid and aortic bodies, i.e., by peripheral chemoreceptors, and information from the peripheral chemoreceptors is transmitted to respiratory and cardiovascular centers in the brainstem whose respiratory and cardiovascular neural outputs are regulated. However, recent progress in neurophysiology has clarified that there are hypoxia-sensors not only in the periphery but also in the central nervous system. Hypoxia also affects the vascular system causing atherosclerosis and pulmonary hypertension and impairs blood glucose regulation that also facilitates atherosclerosis. The effects of hypoxia on vital organs and tissues vary depending on the modality of hypoxia exposure, i.e., acute, chronically sustained, or intermittent hypoxia. Although these issues have been vigorously investigated, the underlying mechanisms are yet to be unraveled. Likewise, long-range consequences for organ and tissue functions affected by hypoxia have not been fully elucidated. In the article collection of this Research Topic, a series of studies report the latest and most notable pathophysiological findings that are categorized into four areas: respiratory control, glucose metabolism, pulmonary hypertension, and sympathetic nervous system activation. The articles attempt to clarify many of the unsolved issues summarized below.

## Respiratory Control

When the body is exposed to hypoxia, ventilation is augmented to increase oxygen uptake from the lungs, although ventilation decreases when the level of loaded hypoxia is too severe. To respond to hypoxia, the body must sense the oxygen content. Carotid body chemosensory or glomus cells play a major role in the monitoring of arterial oxygen levels. However, the exact mechanisms of chemosensing and the nature of the sensor have remained elusive. Ortega-Saenz et al. have reviewed the latest experimental findings concerning the role of specialized mitochondria in acute O_2_ sensing by carotid body glomus cells. They have found that activation of glomus cells by hypoxia depends on the generation of mitochondrial signals (NADH and reactive oxygen species) which modulate membrane ion channels to induce depolarization, Ca^2+^ influx, and transmitter release. Hypoxia is also sensed, to an extent, in tissues other than the carotid body, because peripheral chemodenervated animals augment ventilation in response to hypoxic stimulus (Miller and Tenney, 1975). Chen et al. have reported that a non-selective cation channel, transient receptor potential ankyrin 1 (TRPA1), mediates the physiological responses to mild hypoxia (13–15% O_2_), including avoidance behavior in the hypoxic environment and the wake-up response to hypoxic gas inhalation. They suggested that TRPA1 channels in the sensory nerves innervating the airways detect the hypoxic environment. Recently, it has been reported that medullary astrocytes play a role in central hypoxic sensing (Angelova et al., 2015) with the astrocytic TRPA1 channels as the hypoxia-sensing molecule (Uchiyama et al., 2020). Onimaru et al. have found that some astrocytes in the solitary tract nucleus (NTS) of the dorsal medulla are hypoxia and hypercapnia dual-sensitive. Thus, astrocytes in the NTS may play an essential role in the prevention of hypercapnic hypoxia, which is seen in patients with chronic obstructive pulmonary disease (COPD). Acute hypoxia increases ventilation, and after cessation of hypoxic loading, ventilation decreases but remains above the pre-exposure baseline level for a limited time (Dahan et al., 1995). This post-hypoxic persistent respiratory augmentation (PHRA), i.e., short-term potentiation of breathing, is essential for stabilizing the respiratory output, but the underlying mechanisms have not been well-understood (Eldridge, 1996). Fukushi et al. have investigated this issue and demonstrated that the astrocytes do mediate the PHRA but by mechanisms other than TRPA1 channels that are engaged in hypoxia sensing. These mechanisms are open to conjecture for the time being. Perim et al. have also investigated the issue of respiratory neural plasticity. Moderate acute intermittent hypoxia (mAIH) elicits a progressive increase in the phrenic motor output lasting hours post-mAIH, a form of respiratory motor plasticity known as the phrenic long-term facilitation (pLTF). The authors have demonstrated that elevated PaCO_2_ undermines mAIH-induced pLTF in anesthetized rats, which opposes the known effects of PaCO_2_ on ventilatory long-term facilitation in wakeful humans (Harris et al., 2006). This finding indicates that anesthesia profoundly affects respiratory neural plasticity. It has been postulated that the renin-angiotensin system in the respiratory controller promotes hypoxia-induced chemoreflex sensitization, e.g., by increasing the carotid body sensitivity, and induces central sleep apnea (Fung, 2014). Brown et al. have demonstrated that the severity of hypoxia-induced central sleep apnea is associated with the loop gain, which was assessed by the dynamic ventilatory drive response consequent to a reduction in ventilation due to apnea. However, losartan, an angiotensin-II type-1 receptor (AT1R) antagonist, did not modulate the chemoreflex sensitivity in healthy men before or after nocturnal hypoxia, which sensitizes hypoxia-induced chemoreflex. The contribution of AT1R to the chemoreflex sensitivity in pathological conditions, e.g., heart failure, obstructive sleep apnea, and chronically hypoxic diseases, requires further investigation.

## Glucose Metabolism and Intermittent Hypoxia/Sleep Apnea

Sleep-disordered breathing and intermittent hypoxia often accompany insulin resistance and type 2 diabetes (Koren et al., 2016). Gabryelska et al. have reviewed the role of oxygen-sensitive α-subunit of hypoxia-inducible factor 1 (HIF-1α), a key regulator of oxygen metabolism, as a mediator of insulin resistance and type 2 diabetes in obstructive sleep apnea patients and proposed a concept that stabilization of HIF-1α helps control insulin resistance and diabetes. Concerning the experiments to induce hyperglycemia by loading intermittent hypoxia in animals, Nedoboy et al. have reported that the use of pentobarbital anesthesia is unsuitable because the anesthetic suppresses the intermittent hypoxia-induced blood glucose elevation.

## Pulmonary Hypertension

Hypoxia induces constriction of pulmonary arteries and causes pulmonary hypertension. Fan et al. have investigated the underlying mechanisms of pulmonary hypertension in intermittent hypoxia in rats. They reported that the insulin-induced pulmonary vasodilation and the balance of insulin-induced signaling mediated by upregulated Tribbles homolog 3 (TRIB3), a key mediator of insulin signaling, are impaired in the endothelium, resulting in the development of hypoxic pulmonary hypertension. Umeda et al. have also investigated the mechanisms of chronic intermittent hypoxia-associated atherosclerosis and pulmonary hypertension by evaluating the intima-media thickness (IMT) in the aorta and pulmonary artery in mice. They demonstrated that intermittent hypoxia mimicking obstructive sleep apnea induces IMT thickening that is reversed during the normoxic recovery in both aorta and pulmonary artery. Pulmonary embolism causes hypoxia and may lead to death when the embolus is massive. However, the pathophysiological mechanisms of pulmonary circulation disturbance caused by pulmonary embolism have not been fully elucidated. Wang et al. have investigated the role of inflammatory responses and their relationship to the pulmonary blood flow in the rabbit model of acute pulmonary embolism. The authors suggested that the activation of inflammatory mediators in the tissue around the pulmonary vessels with and without embolus is crucially responsible for pulmonary vascular constriction and pulmonary blood flow attenuation in the model animal.

## Sympathetic Activation

Hypoxia causes excitation of the sympathetic nervous system (Marina et al., 2015; Pijacka et al., 2018). The sympathetic activity is precisely controlled *via* baroreflex. However, the effects of natural hypoxia on the baroreflex function have not been well-understood. Beltrán et al. have investigated this issue in rats placed at a high altitude of 3,270 m above sea level and reported that high altitude produces autonomic and baroreceptor control impairment characterized by parasympathetic withdrawal. High altitude environment in the Himalayas was utilized as a model of hypobaric hypoxia by Verratti et al. The authors compared the delivery of oxygen to muscles in response to resistance exercise between Italian trekkers and native Nepalese porters. The porters had a greater muscle oxygen delivery than Italians, and this ability of porters was attributed to the long-range adaptive memory secondary to frequent exposures to hypoxia. Saha et al. have analyzed the effects of acute hypoxia and hypercapnia on the enhancement of respiratory-sympathetic nerve coupling (respSNA) in the rat model of chronic kidney disease (CKD). The authors demonstrated that female rats with CKD exhibit a heightened respSNA coupling at baseline that is augmented by mild hypoxia but not by hypercapnia. They suggested that this mechanism contributes to driving hypertension in CKD. Postural orthostatic tachycardia syndrome (POTS) is a heterogeneous disease that predominantly affects children and adolescents. POTS patients are markedly sensitized to hypoxia when upright (Taneja et al., 2011). There is a significant difference between children and adults in the diagnosis and treatment of POTS patients. Zhang et al. have classified the clinical forms of POTS into the hypovolemic, neuropathic, and hyperadrenergic POTS, and reviewed the updated individualized treatment strategies for POTS in children. The results may become valuable guidelines in the treatment of POTS.

## Conclusion

Hopefully, the articles published in this Research Topic help readers understand how hypoxia affects the respiratory and cardiovascular systems and how these systems respond to the hypoxic stimulus. A savvy judgment of hypoxia action and adaptation is fundamental for the development of basic research and its translation into improved clinical outcomes in many a disorder, notably hypoxic brain injuries or hypoxic pulmonary pathologies. The understanding of how hypoxia is detected and acts is also essential for psychosomatic and anti-aging rehabilitation strategies to improve the health span. This Research Topic, undoubtedly, reveals the necessity to further investigate the molecular mechanisms of hypoxia-associated cardiorespiratory alterations in health and disease.

## Author Contributions

YO wrote the draft of the manuscript. JP, JL-B, RW, NM, and MP edited the manuscript. All authors contributed to the article and approved the submitted version.

## Conflict of Interest

The authors declare that the research was conducted in the absence of any commercial or financial relationships that could be construed as a potential conflict of interest.

## Publisher's Note

All claims expressed in this article are solely those of the authors and do not necessarily represent those of their affiliated organizations, or those of the publisher, the editors and the reviewers. Any product that may be evaluated in this article, or claim that may be made by its manufacturer, is not guaranteed or endorsed by the publisher.
